# Mitochondrial Protein Network: From Biogenesis to Bioenergetics in Health and Disease

**DOI:** 10.3390/ijms22010001

**Published:** 2020-12-22

**Authors:** Alessandra Ferramosca

**Affiliations:** Department of Biological and Environmental Sciences and Technologies, University of Salento, 73100 Lecce, Italy; alessandra.ferramosca@unisalento.it

Mitochondria are double membrane-bound organelles which are essential for the viability of eukaryotic cells, because they play a crucial role in bioenergetics, metabolism and signaling [1]. Due to their central role in cell life, mitochondria are also involved in the pathogenesis and progression of numerous human diseases [2].

The function of these organelles is mainly regulated by more than 1000 proteins encoded by both mitochondrial and nuclear genomes. Although mitochondria contain an autonomous genome (the mitochondrial DNA), the great majority of mitochondrial proteins are encoded by nuclear genes, synthesized by cytosolic ribosomes, and translocated into mitochondria by a multicomponent import machinery [3,4,5,6].

A large variety of functions have been assigned to mitochondrial proteins, such as respiration, metabolite transport, protein translocation, protein quality control, oxidoreductive homeostasis, and numerous other processes. Interestingly, mitochondrial protein machineries, which have diverse functions, are connected in complex and dynamic networks, and the failure of these systems could lead to the development of disease [1].

The aim of this Special Issue [7] is to reveal the complexity and the versatility of mitochondrial activities, integrating mitochondrial energetics and metabolism with protein biogenesis.

In this context, the review by Watson and McStay [8] offers insights into the functions of cytochrome *c* oxidase (COX) assembly factors. COX and most oxidative phosphorylation complexes are the products of the nuclear and mitochondrial genomes. Therefore, a series of topological and temporal steps must be completed to ensure efficient assembly of the functional enzyme. Without a functional COX enzyme, mitochondria are not able to produce ATP, thus leading to devastating consequences in the context of severely debilitating diseases that often lead to early death.

COX assembly factor 6 (COA6), a small intermembrane space-located protein, plays a role in the biogenesis of COX, as described in an interesting review by Maghool and coauthors [9], which discusses the current understanding of the molecular mechanisms by which COA6 participates in COX biogenesis. In particular, some evidence has indicated that COA6 binds copper, essential for COX assembly, activity, and stability; at the same time, COA6 possesses a thiol oxidoreductase activity, which could correlate with copper binding.

Factors encoded by both nuclear and mitochondrial DNA are involved in the assembly of active respiratory chain complexes and supercomplexes. Interestingly, various nuclear hormone receptors are involved in the regulation of oxidative phosphorylation-related genes. In a review by Kobayashi et al. [10], they focused on the roles of nuclear steroid receptors, including estrogen, estrogen-related, glucocorticoid, mineralocorticoid, progesterone, and androgen receptors in the regulatory mechanisms of mitochondrial respiratory chain complex and supercomplex formation. Clarifying these mechanisms will help to identify therapeutic targets for various diseases, such as heart failure and sarcopenia, where respiratory chain complexes are deeply involved.

In fact, respiratory complexes can exist either individually or organized into the respirasome (a multi-subunit supercomplex of the respiratory chain), and their dynamic interconversion can maintain the structural organization of individual electron transport chain complexes. Regarding this aspect, Chapa-Dubocq et al. [11] examined a cause-and-effect relationship between respirasome depletion and cardiac function in rat intact hearts. Results demonstrated that the disassembly of respirasome by the respiratory complex I inhibitor, rotenone, was associated with diminished cardiac function.

Mitochondrial dysfunction was identified also as an early event of neurodegenerative diseases occurring even before the cognitive deficits. These diseases are characterized by neuronal and/or glial inclusions composed of the microtubule-binding protein tau. Szabo et al. [12] proposed a “mitocentric” picture of tau: this protein was shown to interact with mitochondrial proteins and to impair mitochondrial bioenergetics and dynamics, leading to neurotoxicity.

Among neurodegenerative diseases, Alzheimer’s disease is a multifactorial pathology where alteration of the physical association between the endoplasmic reticulum and mitochondria, also known as mitochondria-associated membranes (MAMs), impacts various cellular functions. Eysert et al. [13] offered an overview of the molecular components of MAMs and their contribution to several paradigms linked to mitochondrial and endoplasmic reticulum physiopathology in general and to Alzheimer’s disease in particular. Potential strategies targeting MAMs to improve mitochondria and endoplasmic reticulum in this neurodegenerative pathology are also suggested.

Mitochondrial dysfunctions can affect any tissue at any age. Zhao et al. [14] discussed a specific subset of diseases caused by pathogenic variants in mitochondrial proteins with interesting skeletal phenotypes, including skeletal dysplasia, skeletal malformations, and metabolic bone disease. Intriguingly, many of these conditions are causally related to factors involved in mitochondrial protein homeostasis, specifically mitochondrial protein import.

Tom70 is a component of the mitochondrial import machinery. In particular, it is a mitochondrial outer membrane protein which is known as a docking site for cytosolic chaperone proteins and co-chaperones. This protein is thereby involved in the uptake of newly synthesized chaperone-bound proteins in mitochondrial biogenesis. In recent years, some studies showed that Tom70 has additional functions, some of them being entirely independent of the biogenesis of mitochondrial proteins. Therefore, this protein was also proposed to be a mediator of membrane contact sites and of innate immunity against viral infections. All these aspects are discussed in an interesting review by Kreimendahl and Rassow [15], which introduces new scenarios to all medical investigations on mechanisms that affect the functions of Tom70 in oncology, neurology, cardiology and immunology.

The understanding of the complexity and the versatility of mitochondrial activities could help also to target the mitochondrial metabolic network as a promising strategy in the treatment of human diseases, such as cancer. In this context, Frattaruolo et al. [16] examined the main mitochondrial metabolic pathways that are altered in cancer, which play key roles in the different stages of tumor progression. Furthermore, they discussed the function of important molecules inhibiting the main mitochondrial metabolic processes, which have been proven to be promising anticancer candidates in recent years.

Not only cancer progression but also mitochondrial stress is a factor that reprograms the mitochondrial biogenesis and metabolism. This topic was examined in the review by He et al. [17]. They analyzed the role of the small ubiquitin-like modifier (SUMO) and its specific proteases as novel mitochondrial stress sensors that respond to the signals produced by various stresses.

Adenosine monophosphate-activated protein kinase (AMPK) is another central mediator of the cellular response to energetic stress and of the mitochondrial homeostasis. Wu and Zou discussed in their review how dysfunctional AMPK contributes to the initiation and progression of cardiovascular diseases via the impact on mitochondrial function [18].

Finally, new perspectives on the understanding of the mitochondrial protein network and its involvement in human mitochondrial diseases could arise from the use of different model organisms. For example, Kleczewska and coworkers [19] reported the biochemical convergence of the mitochondrial Hsp70 system specialized in iron-sulfur (FeS) cluster biogenesis. They clarified the evolutionary relationships between bacterial and mitochondrial Hsp70s specialized in the FeS biogenesis. However, while bacterial proteins are suitable for biochemical studies, the yeast system is an ideal model for in vivo research. Therefore, the similarity between these two systems should help researchers to use them more effectively to gain a better overall understanding of the important process of FeS cluster biogenesis and its linked diseases.

Moreover, Curcio et al. [20] summarized the main features and biological activities of the mitochondrial carriers in *D. melanogaster*, highlighting their similarities and differences with their human counterparts. These proteins are inner mitochondrial membrane proteins which transport different metabolites involved in several biochemical pathways, such as energetic metabolism, hormonal metabolic homeostasis, cell survival, proliferation, and protection from oxidative stress. The characterization of mitochondrial carriers in *D. melanogaster* may result in a better understanding of their physiological roles, also in light of some important insect pathways found to be modulated by these transporters, including fruit fly phenotype, development, fertility, chromosomal integrity, lifespan extension, and survival.

I hope that this Special Issue will provide new research insights and directions in the field of mitochondrial protein networks and their involvement in human health and disease.
